# The Senescent Phenotype of Cultured Hypothalamic Astrocytes from Rats with Streptozotocin-Induced Dementia

**DOI:** 10.1007/s11064-026-04884-7

**Published:** 2026-09-11

**Authors:** Vanessa-Fernanda Da Silva, Daniele Schauren da Costa, Vitor Gayger-Dias, Thomas Michel Sobottka, Ana Carolina Ribeiro Da Silva, Bruna Cristiana R. Biolchi, Laíse Freese da Silva, Nikoli Zasso, Fernanda Becker Weber, Izaviany Schimitz, Diorlon Nunes Machado, Carlos Alexandre Netto, Marina Concli Leite, Larissa Daniele Bobermin, André Quincozes-Santos, Carlos-Alberto Gonçalves

**Affiliations:** 1 Programa de pós- graduação em Bioquímica, Universidade Federal do Rio Grande do Sul (UFRGS), Porto Alegre, Brazil; 2 Programa de pós- graduação em Neurociências, Universidade Federal do Rio Grande do Sul (UFRGS), Porto Alegre, Brazil; 3 Programa de pós-graduação em Biologia Celular e Molecular, Pontifícia Universidade Católica do Rio Grande do Sul (PUCRS), Porto Alegre, Brazil

**Keywords:** Alzheimer’s disease, Astrocyte, Neuroinflammation, SASP, Senescence, S100B

## Abstract

Alzheimer’s disease (AD) is the most prevalent form of dementia, with aging as its primary risk factor. Among experimental models of sporadic AD, intracerebroventricular (ICV) administration of streptozotocin (STZ) in rodents is widely used. We hypothesized that STZ accelerates the process of cellular senescence, which in astrocytes can be assessed using classic markers, including p16, β-galactosidase, Lamin B1, and the senescence-associated secretory phenotype (SASP). To explore this, we cultured hypothalamic astrocytes from adult male Wistar rats at four weeks after STZ-ICV model induction to investigate whether the astroglial changes observed in vivo were maintained under in vitro conditions, and whether markers of senescence were more evident in astrocytes from STZ animals. As in the STZ dementia model, astrocyte cultures exhibited reduced levels of glutamine synthetase and glutathione. Furthermore, elevations in the transcription factors, FOXO3a, Nrf2, and NF-κB, were observed. Markers of unfolded protein response (CHOP and sorcin) were also increased in astrocytes from STZ animals. When evaluating senescence parameters, increases in p53, p21, and p16 were observed, while Lamin B1 levels remained unchanged. With regard to SASP, modulations in TNFα and extracellular HMGB1 and S100B levels also suggested the induction of a senescent profile. Taken together, these findings demonstrate that cultured astrocytes retain key alterations observed in the in vivo model of STZ-induced dementia, highlighting its usefulness for studying AD and therapeutic strategies. Observations in cultures suggest that STZ indeed induces astrocyte senescence and that S100B may be included as a component of the astroglial SASP.

**Supplementary Information:**

The online version contains supplementary material available at https://doi.org/10.1007/s11064-026-04884-7.

## Introduction

Alzheimer’s disease (AD) is the most frequently diagnosed dementia, and accounts for approximately 80% of cases [1]. Histopathologically, AD is characterized by the extracellular accumulation of aggregated misfolded amyloid-β peptide (senile or amyloid plaques) and neurofibrillary tangles of hyperphosphorylated tau protein [2], in addition to astroglial changes that precede and accompany neuronal alterations [3, 4]. Clinically, AD is characterized by memory loss, language impairment, and behavioral changes, leading to functional and social disability [5]. In more than 95% of AD cases, the disease is sporadic, and the main risk factor is aging [6, 7].

Among the methods used to mimic the sporadic model of AD in rodents and primates is the intracerebroventricular administration of streptozotocin, a glucosamine-nitrosourea compound, which initially induces glucose hypometabolism in the central nervous system (pre-amyloid phase) and, within a few months, an increase and deposition of amyloid-β peptides (amyloid phase) [8, 9]. We have used this model to investigate astroglial alterations in both the pre-amyloid and amyloid phases [3, 10, 11].

Although the exact mechanism of action of STZ in the CNS is unknown, this drug is also used to accelerate the appearance of pathognomonic signs of AD (i.e., amyloid plaques) in rodent genetic models of the disease, where it induces the expression of human amyloid precursor protein, which will generate amyloid peptides [12]. Furthermore, cortical astrocyte cultures exposed to STZ have impaired clearance of beta-amyloid peptides and begin producing APP protein [13]. These data reinforce the importance of this model for the study of AD [14].

We hypothesize that STZ may induce cellular senescence, which is characterized by cell cycle arrest, without loss of activity or apoptosis. Cell cycle arrest usually occurs in the G1 phase [15] and is not only related to cell replication, but also to morphological, metabolic, epigenetic, inflammatory changes and the oxidative state of the cell [16]. Senescent astrocytes can be evaluated by changes in general markers such as p16, β-galactosidase, Lamin B1, and senescence-associated secretory phenotype (SASP) [17].

In the central nervous system (CNS), astrocytes are more strongly impacted than neurons by transcriptional changes induced by aging (see [18] for a review). Although behavioral and cognitive changes are more related to changes in the hippocampal and neocortical regions, the greatest susceptibility to astroglial aging is observed in regions such as the hypothalamus and cerebellum. For this reason, it seems reasonable to evaluate STZ-induced senescence in hypothalamic astrocytes.

The STZ-induced Alzheimer’s-type dementia model presents persistent astroglial changes lasting for months. It is possible that these changes would persist if astrocytes were isolated and cultured, expanding the possibilities for study and even therapeutic intervention. In the present study, we induced the dementia model and then isolated and cultured hypothalamic astrocytes, attempting to observe any persistent in vitro changes induced by STZ and confirm our hypothesis of senescence caused by this compound.

## Methodology

### Animals

Forty male 90-day-old Wistar rats (weight: 266 –383 g), obtained from the local breeding colony (CREAL-UFRGS), were transported and maintained in the Biochemistry department of the Universidade Federal do Rio Grande do Sul (UFRGS), Porto Alegre, Brazil, where the experiments were carried out. Animals were maintained under standard conditions, in a 12 h light/dark cycle, at a temperature of 24 ± 2 °C, and 50–60% relative humidity, with free access to water and food. All animal experiments were performed in accordance with the National Institutes of Health (NIH) Guide for the Care and Use of Laboratory Animals and were approved by the Animal Care and Use Committee of the Universidade Federal of Rio Grande do Sul (project number 41744).

### Experimental Design

**Fig. 1:**
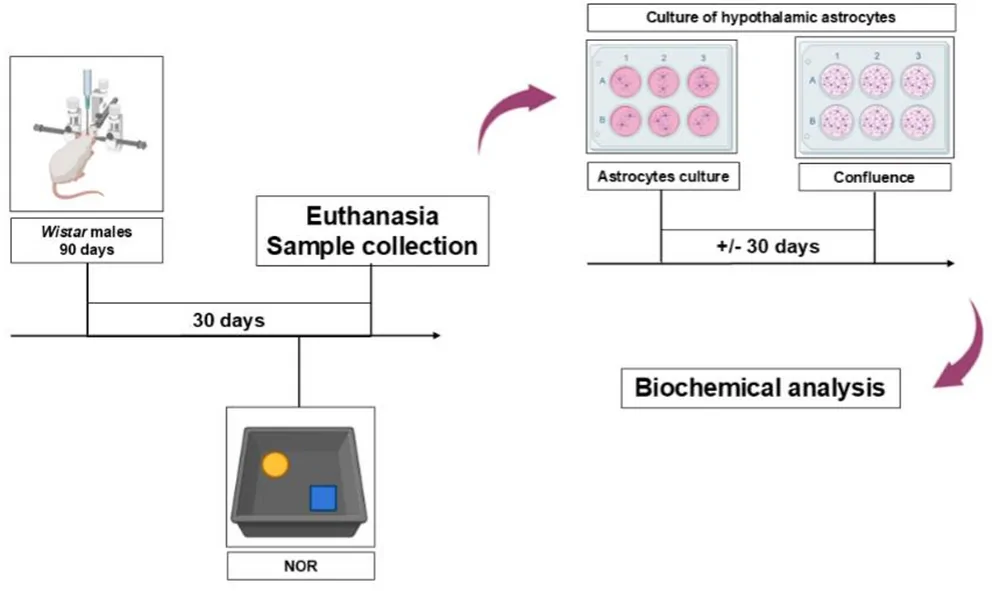
Experimental design. Adult (90-day-old) male Wistar rats were subjected to the Alzheimer’s type dementia model using intracerebroventricular (ICV) infusion of streptozotocin (3 mg/kg), or vehicle. Four weeks later, they were cognitively analyzed by the novel object recognition (NOR) test and subsequently euthanized. Hypothalamus were removed for astrocyte culture. Source: author’s own work, BioRender

In this study, we used male Wistar rats that were subjected to a sAD model induced by STZ-ICV. At twenty-seven days after surgery, the animals underwent the recognition task. On day 30, the animals were briefly anesthetized with isoflurane and decapitated. The brains were dissected in a laminar flow hood, and primary hypothalamic astrocyte cultures were performed as described below. After culture confluence (approximately 30 days), the samples were lysed with buffers specific for each technique, and the samples were frozen in a -80 °C freezer until further analysis. The animals were randomly divided into two experimental groups: control and STZ (Fig. 1).

### Novel Object Recognition (NOR) Task

The behavioral assessment employed was the NOR task, a straightforward memory evaluation that relies primarily on the rodent’s natural exploration tendencies. The task consisted of three phases: habituation, training, and testing. All procedures were carried out during the animals’ active cycle, in an open-field apparatus measuring 50 cm x 50 cm. The animals were acclimated to the testing room for 1 h before the start of each phase of the experiment. On the first day, habituation was conducted, during which the animal was placed facing away from the center of the exploration apparatus for 10 min. On the second day, during the training phase, two identical objects (object A) were positioned diagonally in the peripheral area of the apparatus, allowing the animal to explore them for 5 min. After one hour, the short-term memory (STM) test was administered, in which one of the objects A was replaced with a novel object (object B), keeping the same location, with an exploration time of 5 min. On the third day, 24 h after training, the long-term memory (LTM) test was carried out, where object B was substituted with another new object (object C). The Recognition Index was determined using the following formula: (exploration time of the new object × 100) / (total exploration time of both objects) [3, 11].

### Hypothalamic Astrocyte Culture

The hypothalami of two rats from the same group were removed in a laminar flow hood, and the preparation of the hypothalamic astrocyte cultures was based on previous protocols [19, 20]. The astrocytes were seeded (2–4 × 105 cells/cm^2^) in culture dishes that were pre-coated with poly-L-lysine and cultured in DMEM/F12 (supplemented with 10% fetal bovine serum (FBS), 15 mM HEPES, 14.3 mM NaHCO_3_, 2.5 mg/ml amphotericin B, and 0.05 mg/ml gentamicin) at 37 °C in an incubator with 5% CO_2_. After 24 h, the culture medium was changed and, subsequently, replaced once every 3 days, until the cells reached confluence (approximately 30 days).

### SASP Secretion by ELISA

To ensure the consistency of our results, the culture medium was replaced and, after 1 h, the conditioned medium was collected for Enzyme Linked Immunosorbent Assays (ELISA) analysis of the following secreted factors: BDNF, IL-6, S100B, and TNFα.

ELISAs were performed to evaluate the intra and extracellular levels of BDNF (Millipore Corporation, ref.: CYT306); HMGB1 (ELK Biotechnology, ref.: ELK1439); S100B (ELK Biotechnology, ref.: ELK2613); TNFα (Thermo Fischer Scientific, ref.: 88-7340). All procedures were carried out following the manufacturers’ instructions. The results are expressed as percentages of the control group.

For GFAP levels, ELISA was performed with some modifications, as described by [21]. Briefly, samples were plated onto 96-well high-binding microplates and incubated overnight at 4 °C. Samples were previously diluted in PBS. A standard curve ranging from 0.1 to 10 ng of human GFAP was supplemented with bovine serum albumin (BSA) to give a final quantity of 100 ng/well (diluted in PBS). The plate was blocked with 5% BSA diluted in PBS for 2 h at room temperature. After blocking, anti-GFAP antibody diluted 1:1000 (polyclonal-DAKO, Z0334) in 0.5% BSA/PBS was added and incubated for 1 h at 37 °C. The secondary peroxidase-conjugated anti-rabbit antibody, diluted 1:1000, was then added for 1 h at 37 °C. Subsequently 0.2 mL of peroxidase substrate (Sigma Fast OPD) were added and incubated for 30 min in the dark. The reaction was stopped by the addition of 50 uL 3 M HCl and the absorbance was read at 492 nm on a Thermo Scientific Varioskan Lux spectrophotometer. Three washes were performed with 0.05% Tween-20, diluted in PBS, between each step. The results are expressed as percentages of the control.

### Western Blotting

To perform Western Blotting, astrocyte culture samples were previously homogenized in 4% SDS Stopping Solution (Stopping A) and boiled at a temperature of ≥ 95 °C for 5 min, to denature proteins. After this procedure, the samples were centrifuged at 10,000 X g for 10 min, and the supernatants were collected for the following steps. From the aliquot, 5 µL were used in duplicate for the total protein dosage, using Peterson’s modified Lowry’s method [3]. Glycerol Sample Dilution Solution (40%; Stopping B) was then added to the remaining volume of the sample, before vortexing and storage in a -20 °C freezer until further analysis. After the quantification of total proteins, equal amounts of each tissue sample (20–30 µg), were separated by SDS polyacrylamide gel electrophoresis. Separated proteins were transferred to a 0.45–0.2 μm nitrocellulose membrane. The transfer procedure was confirmed with Ponceau-S staining. Membranes were blocked with a solution of 2% BSA in TBST for 2 h at room temperature (RT). After blocking, membranes were incubated with the corresponding primary antibodies overnight at 4 °C, followed by the respective secondary antibodies conjugated to peroxidase for 2 h at RT (see Table 1). Following secondary antibody incubation, the membranes were stored in TBS for the reading procedure. Between each step, washes with TBST were performed. The nitrocellulose membranes were reused after hydrogen peroxide stripping for different primary antibodies. The chemiluminescence signal was detected using ECL Super Signal West Pico Plus (Thermo Fisher) in a Chemidoc MP (Bio-Rad Laboratories). The optical densities of the images obtained were calculated with the ImageLab software (Bio-Rad Laboratories), and results were normalized using β-Actin density and presented as percentage of the control group.

### RNA Extraction and Quantitative RT‑PCR

Total RNA was isolated from astrocyte cultures using TRIzol Reagent, and the concentration and purity of the RNA were determined spectrophotometrically at a ratio of 260:280 nm using Biodrop. One µg of RNA was then submitted to cDNA synthesis using the High-Capacity cDNA Reverse Transcription Kit (Applied Biosystems/Thermo Fisher Scientific). Quantitative determination of the messenger RNA (mRNAs) targets was performed using the TaqMan real-time RT-PCR system, with inventory primers and probes purchased from Applied Biosystems (Thermo Fisher Scientific), as described in Table 2. Target mRNA levels were normalized to β-actin levels, and the results were analyzed employing the 2^−ΔΔCt^ method [11].

### Immunofluorescence

Cell cultures were incubated with 4% paraformaldehyde for 20 min, permeabilized with 0.1% Triton X-100 in PBS for 5 min at room temperature, and then blocked with 5% BSA for 45 min. Once blocked, cells were incubated overnight with anti-p53 antibody (DAKO, ref: M7001) diluted 1:250 in PBS at 4 °C, followed by washes with PBS and incubation with a specific secondary antibody conjugated with Alexa Fluor^®^ 555 (1:1000) for 1 h at room temperature. The primary antibody was omitted for all negative immunofluorescence control responses. No reactivity was observed when the primary antibody was excluded. Cell nuclei were stained with 0.2 mg/ml 4’,6’-diamino-2-phenylindole (DAPI). Astrocytes were spotted and imaged with a Nikon-specific TE-FM Epi-Fluorescence accessory [22].

### GSH Content Measurement

GSH content was determined as previously described [23]. Briefly, the astrocyte cultures were homogenized in a sodium phosphate buffer (0.1 M, pH 8.0) containing 5 mM EDTA, and protein was precipitated with 1.7% meta-phosphoric acid. Supernatant was assayed with o-phthaldialdehyde (1 mg/mL of methanol) at room temperature for 15 min. Fluorescence was measured using excitation and emission wavelengths of 350 and 420 nm, respectively. A calibration curve was performed with standard glutathione solutions (0–500 mM). The GSH concentrations are expressed as nmol/mg protein [24].

### Protein Content

Total protein content was determined by the Peterson modification of the Lowry method, as previously described [25], using BSA as standard [24].

### Statistical Analysis

The animals were randomly assigned to experimental groups using an online random number generator. Descriptive statistical analysis and assessment of data normality were first performed using the Shapiro–Wilk test. Considering that all data were parametric, the results are presented as means ± standard deviation (SD). For NOR analysis, the one sample T test was used to evaluate differences between the Recognition Index from a chance performance of 50%. For other data, differences between Control and STZ groups were statistically analyzed using Student’s t test, and the value of *p* < 0.05 was used as a criterion for statistical significance. All analyses were performed using GraphPad Prism 9 (GraphPad Software, Inc., La Jolla, CA, USA).

## Results

### Primary Cultures of Astrocytes from Rats with STZ-Induced Cognitive Impairment

To confirm the validity of the dementia model, we performed a NOR task on rats that had received STZ via ICV four weeks previously. Our results confirm the successful induction of the sAD model whereby, in contrast to the control group, the STZ group demonstrated no significant preference for the novel object when compared to the 50% chance level, both at 1 h and at 24 h. This lack of preference at both timepoints indicated short-term memory impairment (Supplementary Fig. 1A, control *p* = 0.0017, STZ *p* = 0.1427) and long-term memory impairment (Supplementary Fig. 1B, control *p* = 0.0016, STZ *p* = 0.1059), respectively.

### Characterization of Cultured Astrocytes from STZ Rats

**Fig. 2:**
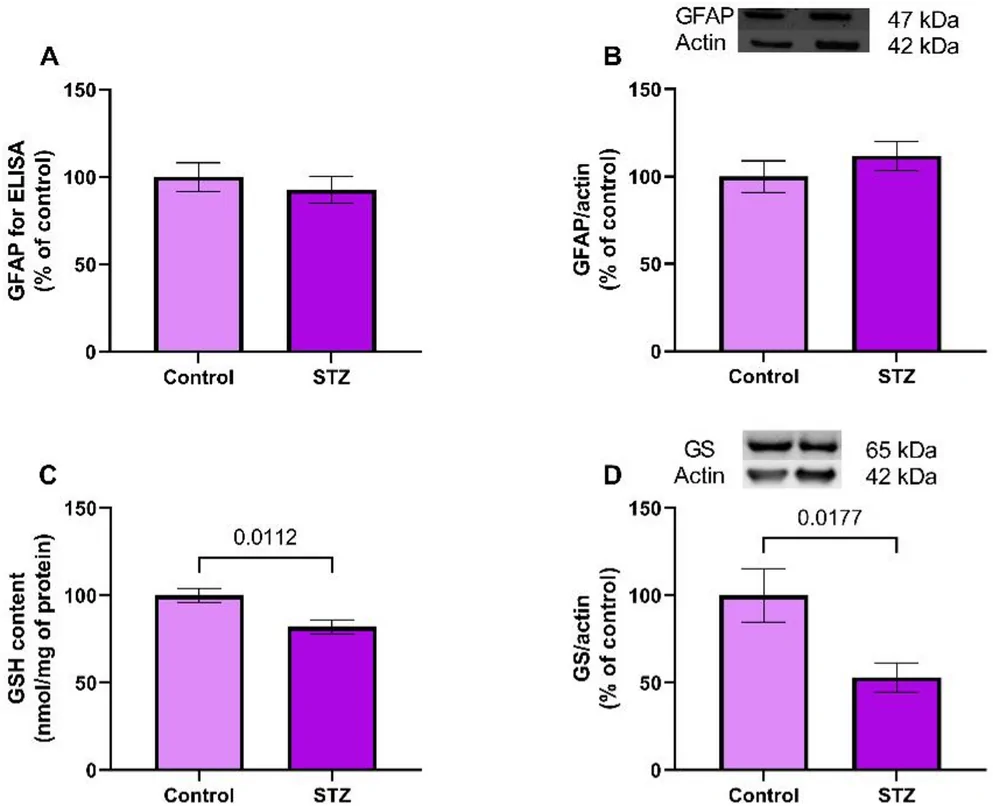
Astroglial markers in primary cultures of animals treated with STZ. GFAP content measured by ELISA in **A** and Western blotting in **B** of hypothalamic astrocytes from STZ-ICV rats; Content of reduced glutathione (GSH), measured by OPT assay in **C**; and glutamine synthetase (GS) content measured by Western blotting in **D**. All data are represented as percentages relative to the control group (*n* = 6). Groups were compared using Student’s t-test, and a p-value < 0.05 was considered statistically significant

We initially assessed the purity of the astroglial cultures after confluence. Over 95% of the cells were positive for GFAP. No quantitative difference in GFAP was observed between the STZ animal cultures and control animal cultures, when evaluated both by Western blotting (Fig. 2B, *p* = 0.3526) and by ELISA (Fig. 2A, *p* = 0.5282).

To assess the activity of cultured astrocytes, we quantitated the glutamine synthetase (GS) enzyme, which is responsible for glutamine synthesis, as well as the glutathione (GSH, an antioxidant) produced. We found a decrease in the amount of GSH in cultures from STZ animals (Fig. 2C, *p* = 0.0112), as well as a reduction in the amount of GS in hypothalamic cultures from STZ animals (Fig. 2D, *p* = 0.0177). Considering these changes in GS and GSH, we also evaluated the glutamate transporters, GLAST and GLT-1 (Supplementary Fig. 2A, *p* = 0.1200 and Supplementary Fig. 2B, *p* = 0.7815, respectively), and observed no changes in the quantities of these transporters.

### Cultured Astrocytes from STZ Animals Exhibit a more Senescent Profile

**Fig. 3:**
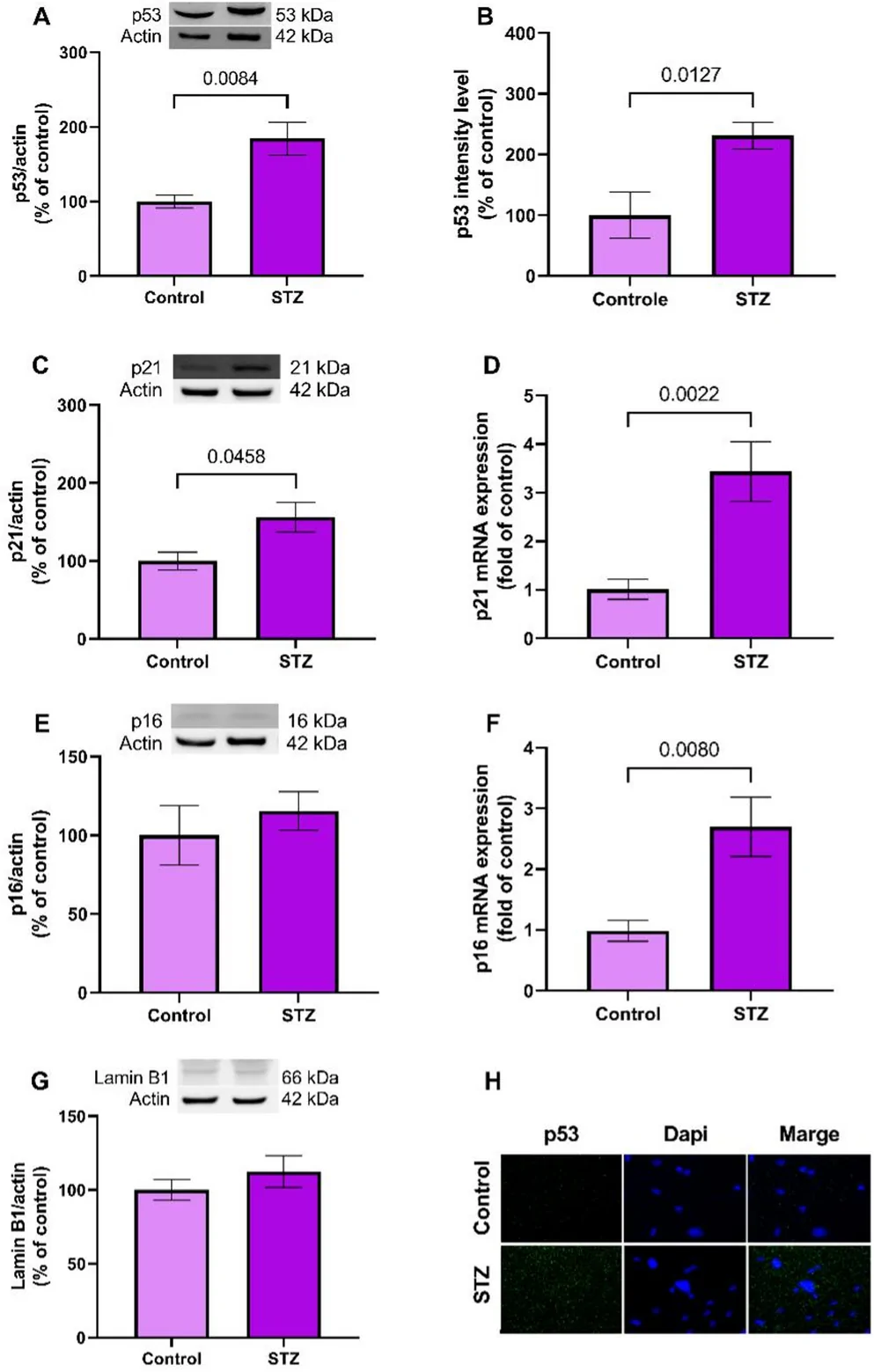
Senescence markers in hypothalamic astrocyte cultures from STZ-ICV rats. Content of p53 measured by Western blotting in **A** and by immunofluorescence in **B** of control and STZ astrocytic cultures; content of p21, measured by Western blotting in **C**, and gene expression, measured by RT-qPCR in **D**; content of p16, measured by Western blotting in **E**, and gene expression, measured by RT-qPCR in **F**; Lamin B1 content, measured by Western blotting in **G**; Panel **H**, representative images of immunofluorescence for p53, with DAPI and merged. Data are represented as percentages or fold relative to the control group (*n* = 5–6). Groups were compared using Student’s t-test, and a p-value < 0.05 was considered statistically significant

Cellular senescence leads to an increase and accumulation of specific cell cycle arrest markers, as well as nuclear alterations. As such, we evaluated the gene expression and/or protein content of p53, p21, p16 and Lamin B1. The p53 protein content was increased in astrocyte cultures from STZ-treated animals (Fig. 3A, *p* = 0.0084 and Fig. 3B, *p* = 0.0127), as shown by western blotting and cellular immunofluorescence, respectively. Protein p21 exhibited an increase in content and gene expression in STZ animal cultures (Fig. 3C, *p* = 0.0458 and Fig. 3D, *p* = 0.0022). In contrast, p16 protein content was not altered in STZ animal cultures compared to control controls; however, a significant increase in gene expression was observed (Fig. 3E, *p* = 0.5013 and Fig. 3F, *p* = 0.0080). Furthermore, we also found no difference in Lamin B1 protein content in the astrocyte cultures from STZ animals (Fig. 3G, *p* = 0.3601). The image in Fig. 3H is representative of the immunofluorescence assays of astrocytic cultures for p53 (quantified in Fig. 3B).

### S100B Secretion is Increased in Senescent Astrocytes

**Fig. 4:**
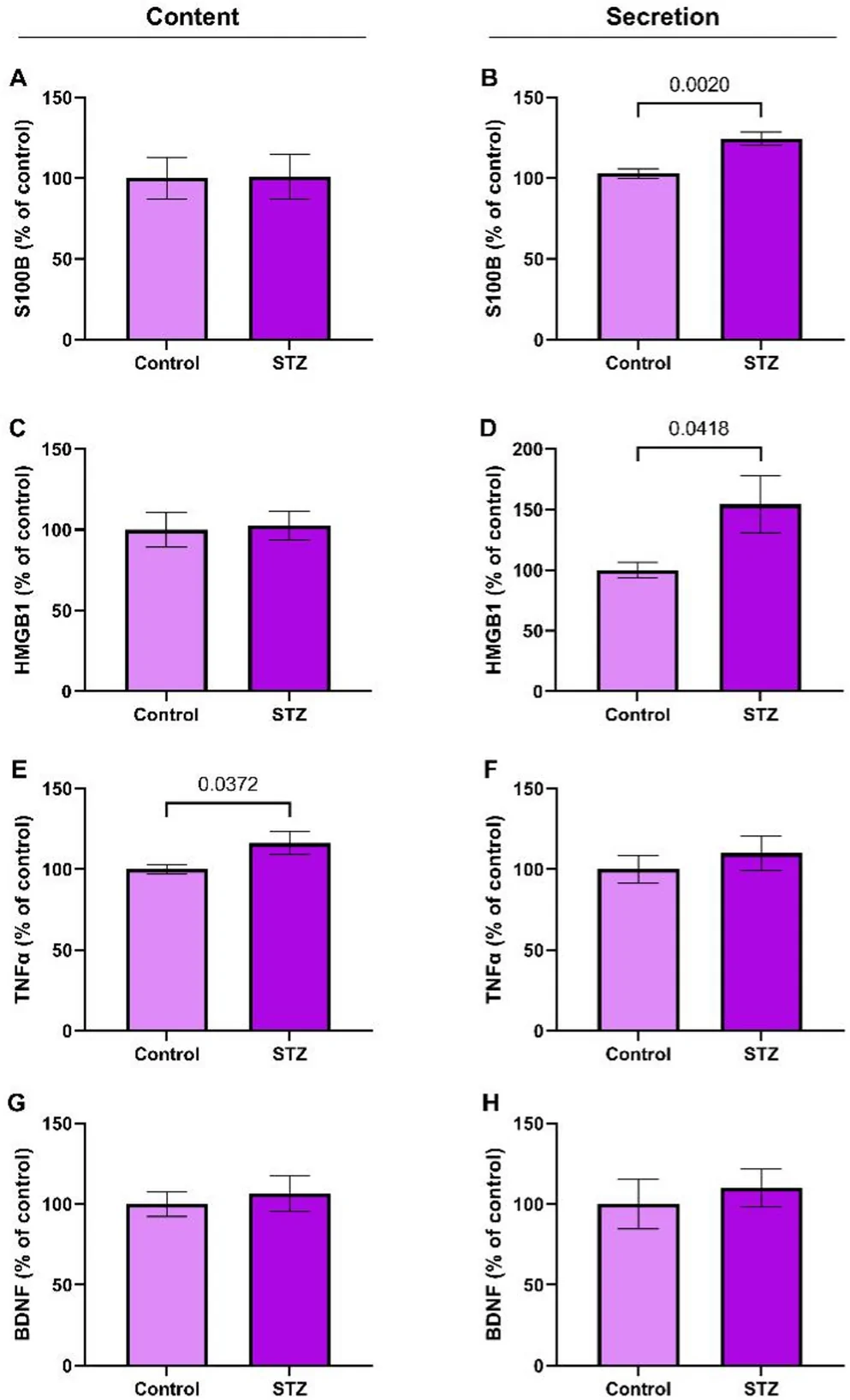
Components of the secretome of cultured hypothalamic astrocytes obtained from ICV-STZ rats. Intracellular in **A** and extracellular in **B** S100B content of control and STZ astrocytic cultures; intracellular in **C** and extracellular in **D** HMGB1 content; intracellular **E** and extracellular **F** TNFα content; intracellular **G** and extracellular **H** BDNF content. All contents were determined by ELISA and data are represented as percentages relative to the control group (*n* = 5–6). Groups were compared using Student’s t-test, and a p-value < 0.05 was considered statistically significant

In addition to general senescence markers, we investigated the expression of some of the astrocytic Senescence-Associated Secretory Phenotype (SASP) proteins. The intracellular content of S100B, a specific astroglial protein, was not increased in cultures from animals treated with STZ (Fig. 4A, *p* = 0.9665), however a significant increase in basal secretion was observed at 1 h (Fig. 4B, *p* = 0.0020). Similarly, the intracellular content of High Mobility Group Box 1 (HGMB1) protein, considered a ubiquitous alarmin, was not increased in astrocytic cultures from animals treated with STZ (Fig. 4C, *p* = 0.8740), however, its basal secretion was increased at 1 h (Fig. 4D, *p* = 0.0418). In contrast, the intracellular content of the TNFα cytokine was increased in astrocyte cultures from STZ animals (Fig. 4E, *p* = 0.0372), but basal secretion at 1 h was not altered (Fig. 4F, *p* = 0.4813). On the other hand, neither the intracellular content nor the basal secretion of the neurotrophin, BDNF, was altered in astrocyte cultures from STZ animals (Fig. 4G, *p* = 0.6273; Fig. 4H, *p* = 0.6190, respectively).

### UPR Proteins are Elevated in Astrocyte Cultures from STZ Animals

**Fig. 5:**
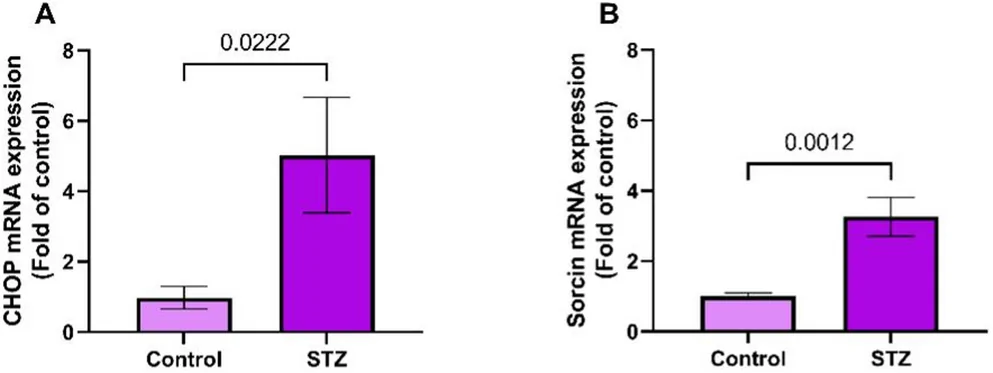
Unfolded protein response markers in hypothalamic astrocyte cultures from STZ-ICV rats. Gene expression of CHOP in **A** and sorcin in **B** in control and STZ astrocytic cultures. Data are represented as fold relative to the control group (*n* = 5–6). Groups were compared using Student’s t-test, and a p-value < 0.05 was considered statistically significant

To evaluate the unfolded protein response (UPR), commonly associated with aging and neurodegenerative disorders, we assessed the gene expression of sorcin and CHOP in astrocyte cultures from STZ animals. The expression of CHOP was increased 5-fold in astrocyte cultures from STZ animals (Fig. 5A, *p* = 0.0222), while sorcin expression increased by approximately 3-fold (Fig. 5B, *p* = 0.0012).

### The FOXO3a Expression, as well as NFκB and Nrf2, is Increased in Senescent Astrocyte Cultures

**Fig. 6:**
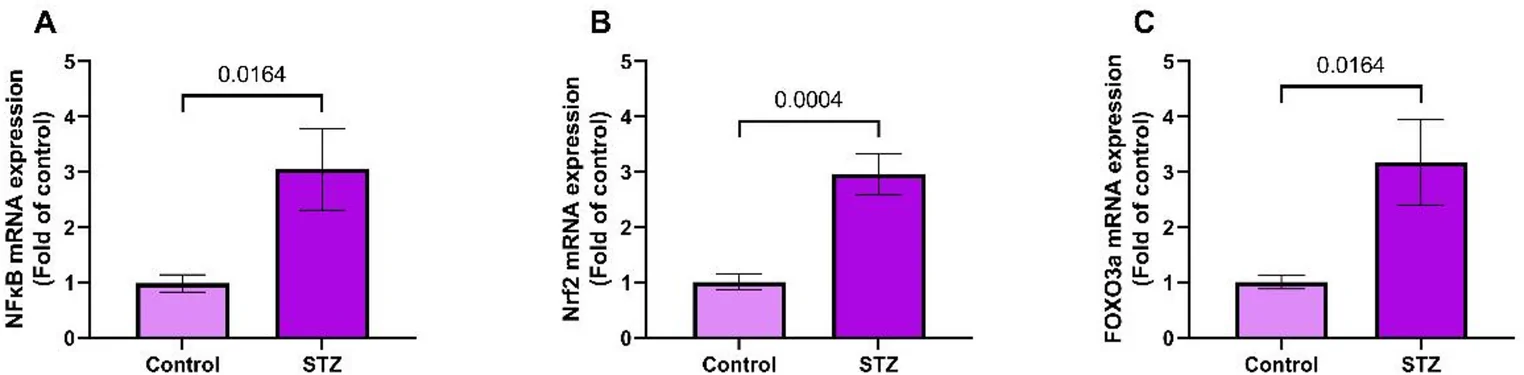
Transcription factors associated with glial reactivity in hypothalamic astrocytes from STZ-ICV rats. Gene expression of NFκB in **A**, Nrf2 in **B**, and FOXO3a in **C** in control and STZ astrocytic cultures. All measurements were determined by RT-qPCR and data are represented as fold relative to the control group (*n* = 5–6). Groups were compared using Student’s t-test, and a p-value < 0.05 was considered statistically significant

The expression of FOXO3a is increased in senescent astrocyte cultures. To evaluate inflammaging, we investigated the gene expression of 3 transcription factors: NFκB, Nrf2, and FOXO3a. An approximately 3-fold increase in the expressions of NFκB (Fig. 6A, *p* = 0.0164), Nrf2 (Fig. 6B, *p* = 0.0004), and FOXO3a (Fig. 6C, *p* = 0.0164) were observed in astrocyte cultures from STZ-treated animals.

## Discussion

As the world’s population ages, the prevalence of Alzheimer’s disease (AD) is increasing [26]. Although AD shares several characteristics with normal brain aging [27], aging remains its greatest risk factor [28], highlighting the need to investigate new pharmacological strategies. In vivo administration of STZ has been widely used as a sporadic model of AD [3, 29–31], and more recently STZ has been used for in vitro models [13]. This study is the first to investigate changes in astrocyte cultures obtained from animals subjected in vivo to the Alzheimer’s-type dementia model. The aim was to evaluate changes in senescence induced in vivo and maintained in vitro. Our findings indicate that the use of this model for studying the disease in vitro is promising and may facilitate the development of therapeutic intervention strategies.

Using primary hypothalamic astrocytes isolated four weeks after ICV-STZ administration, we investigated the relationship between cellular senescence and sAD, as well as a potential astrocyte-specific SASP. Cognitive impairment in the NOR test confirmed successful induction of the sAD model, consistent with previous reports [3, 8]. How aging remodels the CNS remains incompletely understood. The hypothalamus is particularly relevant because it regulates systemic metabolism and the HPA axis [18, 32]. Unlike other brain regions, it appears to exhibit limited astrocyte heterogeneity [33–37], suggesting that hypothalamic astrocytes may play a key role in aging and immunosenescence.

Astrocyte morphology assessment by simple phase contrast microscopy (data not shown) demonstrated no difference between astrocytic cultures of animals treated with STZ and control cultures. Similarly, no difference was observed in GFAP content in the STZ cultures, in contrast to the increase in GFAP observed in animals treated with STZ [3, 29]. Whether this reflects the model (in vivo vs. in vitro) or simply a regional response (hippocampus vs. hypothalamus) is not known at this time. However, both the GSH content, produced mainly by astrocytes [38, 39], and GS protein expression, which is specific to astrocytes in the brain tissue, were lower in hypothalamic astroglial cultures from animals treated with STZ, consistent with observations in the hippocampus of animals subjected to STZ dementia models [10, 40, 41].

Considering specific markers of senescence, we evaluated p53, known as the guardian of the genome [42, 43]. This protein’s activity is strongly regulated by phosphorylation, which modulates its translocation to the nucleus [44–46]. We found an increase in p53 protein content in STZ animal astrocyte cultures, but we lack information about the phosphorylation or location of this protein. When we evaluated p21 in cultures from STZ animals, we also observed a substantial increase in both protein content and gene expression. Moreover, we observed an increase only in the expression of the p16 gene and not in its protein content, possibly because we are observing an early stage of senescence, occurring in STZ animals in the pre-amyloid phase of AD [3, 8].

When cellular senescence sets in, significant nuclear changes occur, including alterations in nuclear size and shape, as well as the formation of micronuclei [47], senescence-associated heterochromatin foci [48], and a decrease in Lamin B1 protein [49]. We herein evaluated the Lamin B1 protein content and observed no significant changes in astrocytes cultured from STZ-treated rats. Notably, a decrease in Lamin B1 is generally associated with an increase in the p16 tumor suppressor [49, 50]. In our study, p16 protein content also remained unchanged in the STZ-animal cultures, consistent with our findings for p53, p21, and sorcin, and suggestive of early changes in cellular senescence.

Senescent cells exhibit a cell-specific SASP [51]. We observed an increase in the extracellular medium content of S100B and HMGB1, with no change in intracellular content, suggesting that these proteins are secreted and may be part of the astrocytic SASP induced by STZ. Similarly to heat shock protein 70 (HSP-70), HMGB1 is a ubiquitous alarmin, while S100B is specific and secreted by astrocytes, working as an alarmin or cytokine [52–54]. We also evaluated the intracellular and extracellular levels of BDNF and TNFα, and observed no changes in BDNF, while TNFα was increased only in the cells. In addition to the classical senescence markers observed, the increase in extracellular S100B in astrocytes from rats exposed to STZ suggest that it is a component of the astrocytic SASP.

Metabolic shifts are directly linked to cellular senescence and, consequently, increases in ROS and RNS [55, 56], which can lead to endoplasmic reticulum (ER) stress [57]. Our group confirmed that the ICV-STZ model of dementia induces alterations in the UPR system in the hippocampus of animals, both at 4 and 16 weeks after model induction [11]. The UPR system plays a role in a complex and not yet understood sequence of events that are triggered by ER stress [58]. Accordingly, increased expressions of the proteins, C/EBP homologous protein (CHOP), which triggers apoptosis, and sorcin, a calcium-binding protein that neutralizes reticular calcium efflux [59, 60] were observed in astrocytes cultured from STZ animals, inferring the presence of ER stress. Interestingly, proteomic analysis of the cerebral cortex from AD patients demonstrated that sorcin is overexpressed, when compared to control individuals, especially in glial cells [61–63]. The ER is the main reservoir of cellular calcium, and ER stress not only increases intracellular calcium levels, but also impairs communication between the ER and mitochondria [57]. The changes in UPR observed indicate the presence of oxidative stress in our cell cultures, suggesting depletion of antioxidant molecules, which is consistent with our findings of decreased GSH. Note that although sorcin has the potential to reduce CHOP expression, in this STZ-induced UPR model, we observed an increase in the gene expression of both proteins [60].

Other notable characteristics of AD include neuroinflammation [2] and an increased redox state [64], which are also observed in the ICV-STZ model [9, 30, 40, 65, 66]. In astrocyte cultures from STZ animals, we observed an increase in the transcription factors, NF-κB and Nrf2, which are responsible for regulating these pathways, respectively. The increase in NF-κB is consistent with our finding of increased intracellular TNFα. Previous studies have demonstrated changes in both NF-κB [67] and Nrf2 [68] in the hippocampus of animals subjected to the same AD model. Interestingly, the FOXO3a transcription factor, whose expression was increased in the STZ-animal derived astrocyte cultures, directly and indirectly modulates the expression of both genes [69–72] and, in astrocytes, regulates the expression of proteins such as aquaporin-4 and GS [73, 74]. Moreover FOXO3a directly regulates the insulin pathway that modulates cell growth and proliferation [72]. Interestingly, the STZ-induced AD model displays glucose hypometabolism and insulin resistance in the CNS [3, 41].

It is important to acknowledge the limitations of this work. Although the changes in the STZ-ICV model are more frequently investigated in the hippocampus (due to its critical role in cognition), our cultures were performed from the hypothalamus tissue because it is highly susceptible to senescence, making it difficult to compare in vivo and in vitro results directly. Nevertheless, despite this limitation, our work provides important findings that improve our understanding of the role of cellular senescence in the STZ-induced AD model, since our cultures presented a persistent senescent phenotype, and aging remains the main risk factor for the development of this disease.

**Fig. 7:**
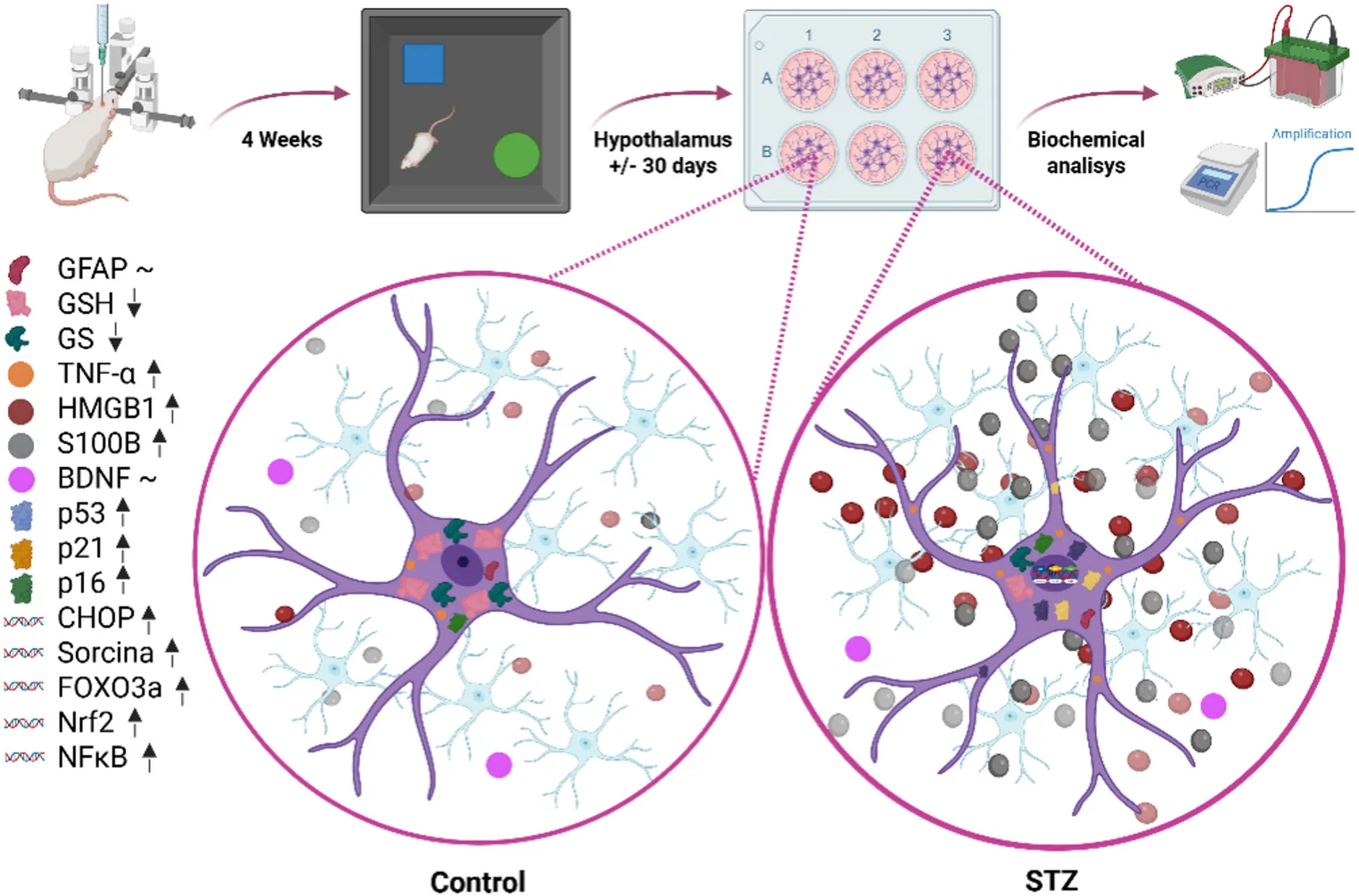
Astroglial parameters in primary cultures of animals treated with STZ. Parameters analyzed are on the left and changes are represented by unchanged (~), increased (↑), or reduced (↓). Source: author’s own work, BioRender

**Table 1:** List of antibodies used

Antibody	% of gel	RRID number	Dilution	Incubation
Anti-GFAP	12	Sigma, G9269	1:1000	1 ON 4 °C
Anti-Glast	12	Abcam, 963,286	1:1000	1 ON 4 °C
Anti-GLT-1	12	Abcam, GR124542-1	1:1000	1 ON 4 °C
Anti-GS	12	Santa Cruz, sc-32,560	1:1000	1 ON 4 °C
Anti-Lamin B1	12	Elabscience, E-AB-20,076	1:1000	1 ON 4 °C
Anti-mouse-HRP	–	Invitrogen, 31,430	1:10000	2 h RT
Anti-p16	16	ELK, ES8349	1:1000	2 ON 4 °C
Anti-p21	16	Elabscience, E-AB-66,357	1:1000	2 ON 4 °C
Anti-p53	12	DAKO, M7001	1:1000	1 ON 4 °C
Anti-rabbit-HRP	–	Elabscience, E-AB-1058	1:10000	2 h RT
Anti-β-actin-HRP	–	Proteintech, HRP600008	1:30000	2 h RT

*GFAP* glial fibrillary acidic protein, *Glast* Glutamate/Aspartate Transporter, *GLT-1* Glutamate Transporter-1, *GS* glutamine sintetase, *HRP* horseradish peroxidase, *ON* overnight, *RT* room temperature, *RT* room temperature

**Table 2:** Genes analyzed by quantitative RT-PCR

mRNA target	Assay ID
β-actin	Rn00667869_m1
C/EBP homologous protein (CHOP)	Rn00492098_g1
CDKN1A/p21^Cip1^ (p21)	Rn00589996_m1
CDKN2A/p16^INK4a^ (p16)	Rn00580664_m1
Forkhead Box O-3a (FOXO3a)	Rn01441087_m1
NF-E2-related factor 2 (Nrf2)	Rn00582415_m1
Nuclear factor kappa B (NFκB)	Rn01399572_m1
Sorcin	Rn01529190_m1

## Conclusions

Our data demonstrate that hypothalamic astrocytes cultured from the ICV-STZ rat model of dementia of the Alzheimer’s type exhibit increased senescence markers, such as p53, p21, and p16, as well as increased markers of oxidative stress and neuroinflammation, such as GSH, Nrf2, NFκB, and TNFα. See a summary of the results in Fig. 7. The increased S100B secretion in these cultures suggests that this protein can be incorporated as a component of the astrocytic SASP. Together, these data show that the cultured astrocytes from these animals preserve these alterations, allowing for a broader understanding of the model and the disease in vitro, for which age is the major risk factor.

## Supplementary Information

Below is the link to the electronic supplementary material.

Supplementary Material 1

## Data Availability

The data used to support the conclusions of this study are available from the corresponding author upon request.
