# Supplementary material for: The Senescent Phenotype of Cultured Hypothalamic Astrocytes from Rats with Streptozotocin-Induced Dementia
Source: Neurochem Res. 2026 Sep 11;51(5):265. doi: 10.1007/s11064-026-04884-7 (PMC13569601; doi:10.1007/s11064-026-04884-7)
Supplement: Supplementary file 1 [file 11064_2026_4884_MOESM1_ESM.docx]

Supplementary Fig. 1. Animals subjected to the Alzheimer’s type dementia model exhibit cognitive deficit at four weeks after STZ-ICV infusion. A) Short-term memory; B) Long-term memory. The behavioral test of NOR (n = 12) was performed. The Recognition Index (RI) was calculated based on the following equation (exploration time of the new object x100/exploration time of both objects). The line on the graph indicates 50%. The data are represented as a percentage referring to the control group. The p-value represents the statistically different group (t-test for one sample, assuming p < 0.05, where p < 0.05 versus chance level – 50% of the investigation of new objects in the discrimination phase)

Supplementary Fig. 2. There are no alterations in glutamate transporters in hypothalamic astrocytes of STZ-ICV rats. Glast/EAAT1 (A) and GLT-1/EAAT2 (B) transporters were measured by Western blotting. All data are presented as a percentage relative to the control group (n = 6). The p-value represents the statistical difference (Student's t-test, considering p < 0.05). Abbreviations: EAAT, excitatory amino acid transporter; Glast, glutamate aspartate transporter; GLT-1, glutamate transporter 1
